# Plasmepsins IX and X are essential and druggable mediators of malaria parasite egress and invasion

**DOI:** 10.1126/science.aan1478

**Published:** 2017-10-27

**Authors:** Armiyaw S. Nasamu, Svetlana Glushakova, Ilaria Russo, Barbara Vaupel, Anna Oksman, Arthur S. Kim, Daved H. Fremont, Niraj Tolia, Josh R. Beck, Marvin J. Meyers, Jacquin C. Niles, Joshua Zimmerberg, Daniel E. Goldberg

**Affiliations:** 1 Division of Infectious Diseases, Department of Medicine, Washington University School of Medicine, Saint Louis, MO 63110, USA; 2 Department of Molecular Microbiology, Washington University School of Medicine, Saint Louis, MO 63110, USA; 3 Section on Integrative Biophysics, *Eunice Kennedy Shriver* National Institute of Child Health and Human Development, National Institutes of Health, Bethesda, MD 20892, USA; 4 Faculty of Biology, Medicine and Health, Division of Infection Immunity and Respiratory Medicine, School of Biological Sciences, University of Manchester, Manchester, UK; 5 Department of Pathology and Immunology, Washington University School of Medicine, Saint Louis, MO 63110, USA; 6 Center for World Health and Medicine, Saint Louis University School of Medicine, Saint Louis, MO 63104, USA; 7 Department of Biological Engineering, Massachusetts Institute of Technology, Cambridge, MA 02139, USA

## Abstract

Proteases of the malaria parasite *Plasmodium falciparum* have long been investigated as drug targets. The *P. falciparum* genome encodes 10 aspartic proteases called plasmepsins, which are involved in diverse cellular processes. Most have been studied extensively but the functions of plasmepsins IX and X (PMIX and PMX) were unknown. Here we show that PMIX is essential for erythrocyte invasion, acting on rhoptry secretory organelle biogenesis. In contrast, PMX is essential for both egress and invasion, controlling maturation of the subtilisin-like serine protease SUB1 in exoneme secretory vesicles. We have identified compounds with potent antimalarial activity targeting PMX, including a compound known to have oral efficacy in a mouse model of malaria.

Considerable work has gone into the synthesis of plasmepsin inhibitors as antimalarials (*1**–**4*). Most efforts have been directed at the digestive vacuole plasmepsins I to IV (PMI to PMIV) because of the availability of crystal structures and recombinant proteins. However, genetic knockouts reveal that these are not essential for parasite survival (*5*). Thus, rationally designed inhibitors of PMI to PMIV likely exert their antimalarial effects through other targets (*1*, *3*). PMV, PMIX, and PMX are the only other plasmepsins expressed in asexual blood-stage parasites (*6*). PMV is an essential protease that processes proteins for export into the host erythrocyte and is a focus of ongoing drug development efforts (*7**–**9*). PMV is, however, quite divergent from the other plasmepsins (*10*), and most digestive vacuole plasmepsin inhibitors are not potent against this enzyme (*11*). PMI to PMIV share more sequence homology to PMIX and PMX (*10*). PMIX and PMX could be the targets of digestive vacuole plasmepsin inhibitors that have antimalarial activity.

To characterize the functions of PMIX and PMX in the biology of blood-stage *Plasmodium falciparum* parasites, we used new TetR-aptamer conditional knockdown (KD) technology (*12*) (fig. S1), enabling translational repression of the target gene when anhydrotetracycline (aTc) is removed from the culture. Using CRISPR-Cas9 editing, we installed the TetR-aptamer regulatory system at the PMIX and PMX loci to create PMIX^apt^ and PMX^apt^ lines (fig. S1). When aTc levels were lowered in synchronous, early ringstage parasites, we observed a major decrease in target protein levels in late-stage schizonts, in both PMIX^apt^ and PMX^apt^ (Fig. 1A). This led to decreased replication, revealing a critical role for both of these enzymes in parasite survival (Fig. 1B).

**Fig. 1:**
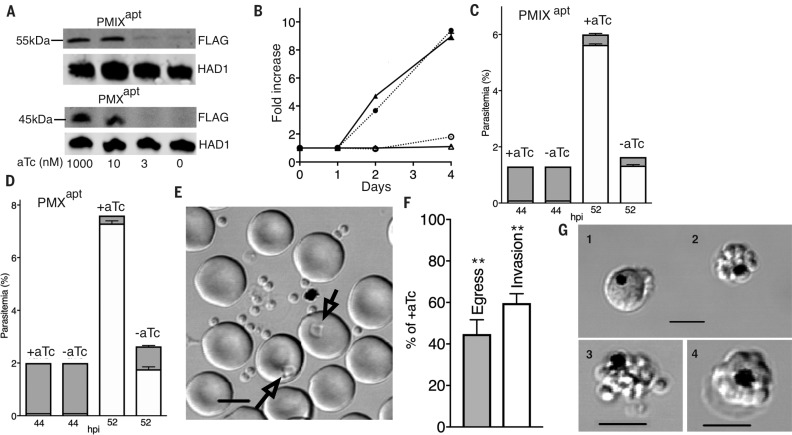
**PMIX is essential for invasion of erythrocytes; PMX is essential for egress and invasion.** (**A**) Immunoblot with aFlag antibody showing knockdown (KD) of PMIX and PMX after anhydrotetracycline (aTc) withdrawal for one cycle. Haloacid dehalogenase–like hydrolase (HAD1) was used as a loading control. (**B**) Expansion of PMIX^apt^ and PMXapt parasites is impaired in –aTc medium (*P* < 0.0001 for each by two-tailed t test). Triangles, PMIX^apt^; circles, PMX^apt^. Open symbols, –aTc; closed symbols, +aTc. (**C** and **D**) PMIX KD results in an invasion defect. PMX KD results in both egress and invasion defects. Synchronized ring-stage cultures were grown with or without aTc. Schizonts and rings were counted by flow cytometry at 44 and 52 hours postinvasion (hpi). Shaded bars, schizonts; open bars, rings. (C) 52-hour rings were fewer in the –aTc condition [*P* < 0.0001 (t test)]. Number of schizonts was not significantly different. (D) 52-hour rings were fewer in the –aTc condition (*P* < 0.0001). Number of remaining schizonts was greater (*P* < 0.001). (**E** to **G**) Live-cell microscopy of PMX parasites with or without aTc. (E) Individual schizonts were scored for egress and subsequent invasion (arrows in image of control parasite field). Scale bar, 5 μm. (F) Quantification of PMX^apt^ egress and invasion defects in the –aTc condition (**P < 0.01). (G) Abnormal schizont classes observed after PMX KD: 1, distorted schizont; 2, unruptured merozoite cluster; and 3, defective egress or merozoite dispersal. 4, Normal schizont for comparison. Scale bars, 5 μM. All experiments in this and subsequent figures were replicated at least three times. +aTc: 1 μM. Error bars indicate SEM.

To determine the stage at which the defect occurred, cell cycle progression was monitored using highly synchronous ring-stage parasites cultured under KD (–aTc) or induced (+aTc) conditions. Flow cytometry revealed that both PMIX^apt^ and PMX^apt^ developed normally until they reached segmented schizonts (~44 hours). At the end of the cycle (between 46 to 52 hours), similar numbers of PMIX^apt^ schizonts had egressed, irrespective of PMIX expression status. However, –aTc cultures featured about one-fourth as many new rings (Fig. 1C). A similar fourfold decrease was seen in –aTc PMX^apt^ cultures (Fig. 1D). Unlike for PMIX^apt^, at 52 hours, 80% of PMX^apt^ parasites had egressed in +aTc cultures, whereas only 36% had egressed in –aTc cultures. Even taking the egress defect into account, however, we observed fewer rings than expected, indicating an additional invasion phenotype. We further characterized this by live microscopy of individual cells (*13*) (Fig. 1E) and observed similar egress and invasion impairment (Fig. 1F). Unruptured schizonts accumulated in the –aTc culture as merozoite clusters and some distorted schizonts; occasionally, these partially ruptured or displayed defective merozoite dispersal (Fig. 1G). Of the parasites that could egress in a normal time frame under –aTc conditions, the merozoite invasion rate was ~50% of that observed in the presence of aTc (Fig. 1F). These data implicate PMIX in erythrocyte invasion and PMX in both egress and invasion. A PMIX^apt^-PMX^apt^ double aptamer– tagged line displayed a similar defect in egress and a greater block in invasion, yielding a sevenfold decrease in new rings (fig. S2). The data imply independent contributions of PMIX and PMX to these processes.

To evaluate the subcellular localization of these proteins, we engineered epitope tags on the 3′ end of the endogenous genes (fig. S3) and performed immuno–electron microscopy. PMIX was found largely in the bulbs of rhoptry secretory organelles that are involved in invasion (*14*) (Fig. 2, A and B). PMX was found in exonemes—small, ovoid secretory vesicles that discharge during egress into the parasitophorous vacuole surrounding the parasite (*15*) (Fig. 2, C to E).

**Fig. 2:**
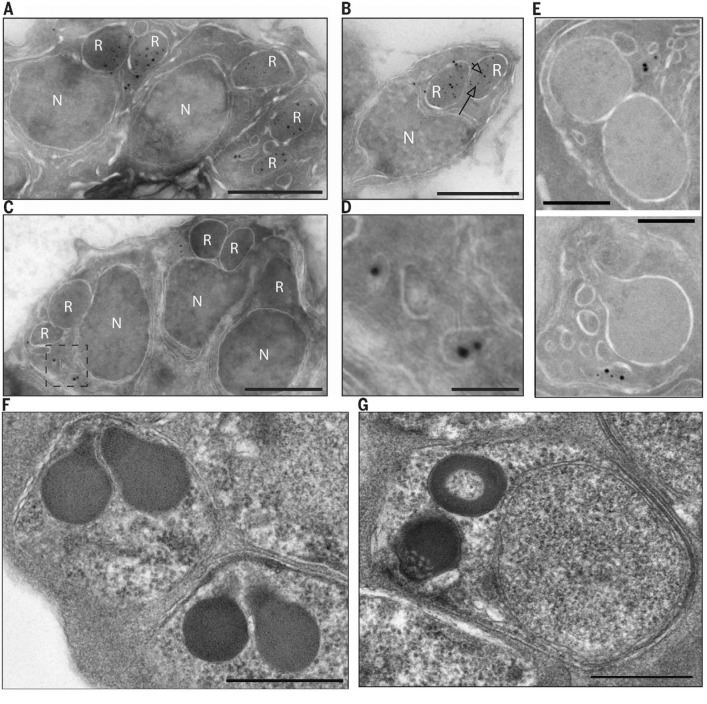
**PMIX localizes to rhoptries, whereas PMX localizes to exonemes.** (**A** and **B**) The PMIX gene was tagged with 3X hemagglutinin (HA) sequence at the 3′ end of the endogenous open reading frame. Tagged parasites grew normally. Segmented schizonts were prepared for immuno–electron microscopy, and PMIX was visualized with an anti-HA antibody and an 18-nm colloidal gold-labeled secondary antibody. Colocalization with an anti-RAP1 antibody, a marker for rhoptries, was performed using a 12-nm colloidal gold-labeled secondary antibody. The arrowhead points to an 18-nm particle; the arrow points to a 12-nm particle. (A) schizont; (B) merozoite. (**C** and **D**) The PMX gene was tagged with HA sequence. (C) Schizont; (D) enlarged view of the boxed area in (C). (**E**) The PMX gene was tagged with green fluorescent protein (GFP) sequence, and the exoneme marker SUB1 was tagged with 3X HA. Segmented schizonts were prepared for immuno–electron microscopy, PMX was visualized with an anti-GFP antibody and an 18-nm colloidal gold-labeled secondary antibody, and SUB1 was visualized with an anti-HA antibody and a 12-nm colloidal gold-labeled secondary antibody. Two examples are shown. Controls omitting the primary antibody were negative in all cases. (**F** and **G**) Electron micrographs of PMIX^apt^ cultured with (F) or without (G) aTc. Note the apical granularity and discoid morphology (G). See fig. S1 for statistics. Scale bars, 500 nm [(A) to (C), (F), and (G)]; 100 nm (D); 200 nm (E). R, rhoptry; N, nucleus.

The localization studies guided us to examine organellar proteins whose processing could be affected by PM action. RAP1 is a rhoptry bulb protein that is processed from an 84-kDa precursor to 82- and 67-kDa forms. The PMIX^apt^ line failed to process the precursor efficiently when aTc was withdrawn (Fig. 3A). In contrast, the rhoptry neck protein RON4 was processed despite PMIX KD. By electron microscopy, a rhoptry biogenesis defect was evident under KD conditions (Fig. 2, F and G).

**Fig. 3:**
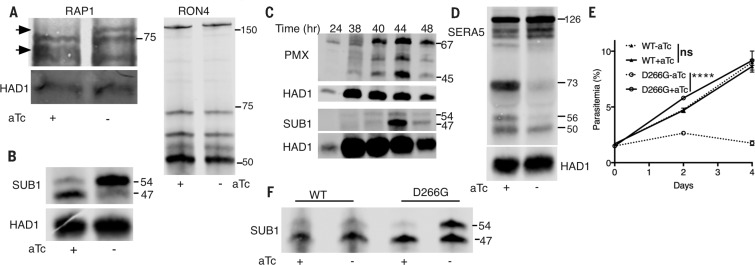
**Plasmepsin knockdowns impair schizont protein maturation.** (**A**) RAP1 maturation is impaired when PMIX is knocked down, but RON4 maturation is not. PMIX^apt^ parasites were cultured with and without aTc to 44 hpi, and extracts were blotted for RAP1 (left) and RON4 (right). Arrows mark precursor and processed RAP1 forms. (**B**) The second maturation step in SUB1 processing is defective when PMX is knocked down. PMX^apt^ parasites were cultured as in (A). The rabbit anti-SUB1 immunoblot shows 54-kDa intermediate and 47-kDa mature SUB1 forms. (**C**) PMX expression starts before SUB1 expression, but SUB1 maturation coincides with the peak of mature PMX. HA-tagged PMX parasites were cultured for varying times. Parasite extracts were processed for immunoblot using anti-HA or anti-SUB1 antibodies. Precursor (PMX, 67 kDa; SUB1, 54 kDa) and mature (PMX, 45 kDa; SUB1, 47 kDa) forms are marked at right. (**D**) Immunoblot shows that processing of the SUB1 substrate SERA5 is impaired by PMX KD. PMX^apt^ parasites were cultured for 46 hpi (with and without aTc) and processed for immunoblot. (**E** and **F**) A catalytically dead mutant cannot rescue PMX KD. PMX^apt^ parasites were complemented with a second copy of the PMX gene, wild-type (WT) or mutant (D266G). Cultures were maintained with and without aTc; parasitemia (E) and SUB1 processing (F) were assessed. ****P < 0.0001 by two-tailed t test. KD of PMX and expression of second-copy genes were confirmed by immunoblot (fig. S6). ns, not significant. Error bars indicate SEM.

The subtilisin-like serine protease SUB1 is an exonemal protein that plays a critical role in egress and invasion (*15*, *16*). SUB1 is synthesized as an 82-kDa zymogen that rapidly self-processes into a 54-kDa semi-proenzyme in the endoplasmic reticulum (ER). The cleaved prodomain remains bound to the 54-kDa protein and inhibits activity (*17*). A second processing step converts the 54-kDa form into a 47-kDa mature protein. This step can occur autocatalytically in vitro but is slow and partial (*18*, *19*). A processing enzyme has been postulated for this step (*20*), but its identity is unclear. Notably, a major defect in SUB1 processing was observed in PMX^apt^ (Fig. 3B) but not PMIX^apt^ (fig. S4) after aTc withdrawal, indicating that PMX is important for the final SUB1 processing step. Consistent with this, PMX is synthesized and processed shortly before SUB1 synthesis and processing occurs (Fig. 3C). Similar to the second processing step of SUB1, PMX maturation is blocked by brefeldin A (fig. S5), suggesting a post-ER event.

During egress, SUB1 processes a family of cysteine proteases (SERAs) and a family ofmerozoite surface proteins (MSPs) (15, 16). SERA5 is synthesized as a 126-kDa protein and is processed sequentially by SUB1 into 73- and 56-kDa forms. The latter is further processed into a 50-kDa fragment in a SUB1-independent process (21). We assessed SERA5 in PMX^apt^ parasites. In the absence of aTc, SERA5 accumulated in the 126-kDa form with very little processing to other intermediates (Fig. 3D). Similarly, MSP1 accumulated as its 193-kDa precursor (fig. S6). Thus, PMX KD impairs downstream egress events.

We tested whether PMX is an active protease by introducing an ectopic gene copy (Fig. 3, E and F, and fig. S7). PMX^apt^ parasites constitutively expressing a second-copy PMX gene with an active site aspartate mutation [Asp^266^→^Gly266^ (D266G)] had reduced growth in the absence of aTc and were unable to restore processing of SUB1. In contrast, those expressing a wild-type second-copy gene were rescued. These data show that PMX enzymatic activity in vivo is crucial to its function. The catalytic mutant PMX was processed to the mature form, suggesting a transprocessing event (fig. S7B).

Many aspartic protease inhibitors with antimalarial properties have been investigated, but the specific targets for most of them are unknown (*1*). Three aminohydantoins that caused schizont accumulation reminiscent of our PMX KD phenotype were identified [TCMD-134675 and TCMD- 136879 from the TCAMS collection (*22*) and CWHM-117 (*11*)]. To evaluate whether these compounds act in a PMIX- or PMX-dependent manner, dose-response curves were determined for KD parasites. The half-maximal effective concentration values were substantially lower for PMX^apt^ but not PMIX^apt^ cultured in low-aTc conditions (Fig. 4, A and B). This indicates hypersensitivity of PMX^apt^ parasites to aminohydantoins when PMX expression is low. Egress and invasion were blocked, and an accumulation of abnormal schizonts was observed by live-cell microscopy as shown with CWHM-117 (Fig. 4, C and D). Further, treatment of parasites with each of these inhibitors blocked SUB1 and SERA5 maturation (Fig. 4E), as was seen with PMX KD. Treatment with aminohydantoins did not block the initial SUB1 autoprocessing step. The compounds also did not inhibit PMX maturation (Fig. 4E). Recombinant PMX cleaved a fluorogenic peptide as well as SUB1 protein in vitro, and these reactions were blocked by aminohydantoins (Fig. 4F and fig. S9), with median inhibitory concentration values from 175 to 800 nM in the peptide assay. Combining PMX KD and inhibitor treatment resulted in a complete block of egress and SUB1 maturation (Fig. 4, G and H). Because CWHM-117 has oral efficacy in a mouse model (*11*), it appears that PMX is a promising target for antimalarial chemotherapy.

**Fig. 4:**
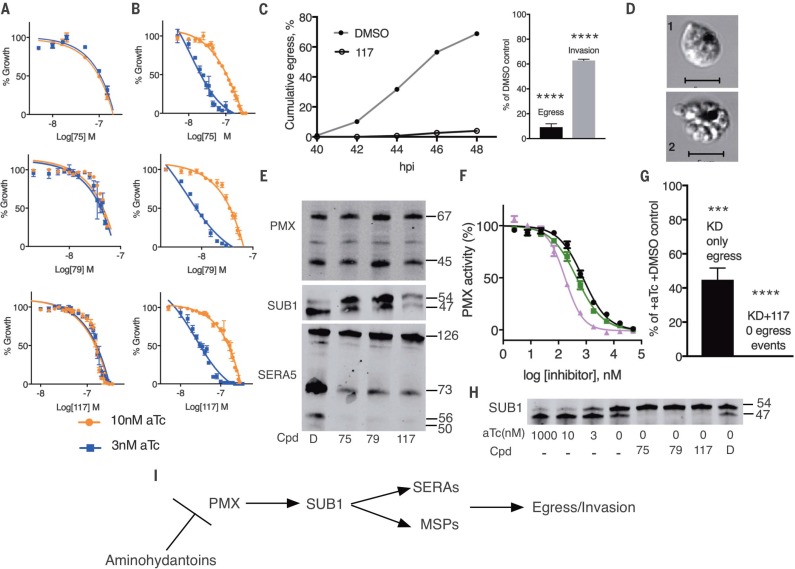
**Aminohydantoin compounds act in a PMX-dependent manner and phenocopy the PMX knockdown through a block in SUB1 processing.** (**A** and **B**) Dose-response curves for compounds incubated for 48 hours with PMIX^apt^ (A) or PMX^apt^ (B) parasites at low (3 nM) or higher (10 nM) aTc concentrations. Values are mean ± SEM (error bars). Growth of parasites was similar at both aTc concentrations (fig. S8). (**C** and **D**) CWHM-117 blocks egress at the same stage as PMX KD. Parental (NF54) parasites were synchronized, treated with 500 nM CWHM-117 from 24 hpi, and monitored for progression of egress by live cell microscopy, as in Fig. 1. (C) Successful egress and invasion was scored. ****P < 0.0001. (D) 1, distorted schizont; 2, merozoite cluster. Scale bars, 5 μM. (**E**) Aminohydantoins block the final SUB1 maturation step and impair processing of downstream substrates. Compounds were applied to PMX-3X HA cultures at 24 hpi and incubated for another 24 hours. Parasite extracts were analyzed for immunoblot using antibodies to SUB1, SERA5, and HA (for PMX). Cpd, compound; D, dimethyl sulfoxide (DMSO) vehicle control; 75, 0.2 μMTCMDC-134675; 79,0.1 μM TCMDC-136879; 117,0.5 μMCWHM-117. (**F**) PMX assay. Recombinant PMX was incubated with fluorogenic peptide for 120 min in the presence or absence of aminohydantoins. Black, CWHM-117; green, TCMDC-136879; pink, TCMDC-134675. Error bars, SEM. (**G** and **H**) PMX KD combined with inhibitor treatment completely blocks egress (****P < 0.0001; ***P < 0.001) (G) and SUB1 maturation (H). Labels and concentrations are as in (E). (**I**) Scheme of proteolytic cascade involved in egress.

We have determined that PMIX and PMX are essential for parasite egress and invasion. PMIX localizes to the rhoptries and could be a maturase for proteins in this organelle.We have discovered thatPMXis required for SUB1 processing,making PMX the most upstream protease known in the egress cascade (Fig. 4I). However, it is not clear that the final cleavage of SUB1 is a direct action of PMX; if so, it could result from processing of semi-pro SUB1 or fromcleavage of the prodomain, liberating semi-pro SUB1 to process itself. PMX is capable of cleaving SUB1 in vitro (fig. S9), adding some support to the direct-cleavage model.

PMX does not appear to autoprocess (Fig. 4E and fig. S7B), unlike most aspartic proteases, which suggests that there may be another stillundiscoveredmaturase upstream in the proteolytic cascade of egress. We have identified compounds with a common scaffold that are specific inhibitors of PMX and that recapitulate the actions of PMX KD phenotypically. Our PMIX and PMX lines should allow high-throughput screening of aspartic protease inhibitor collections andmay inform efforts to improve on the promising CWHM-117 lead compound.

## SUPPLEMENTARY MATERIALS

www.sciencemag.org/content/358/6362/518/suppl/DC1

Figs. S1 to S9

References (23–29)
