# Supplementary material for: Plasmepsins IX and X are essential and druggable mediators of malaria parasite egress and invasion
Source: Science. 2017 Oct 27;358(6362):518–22. doi: 10.1126/science.aan1478 (PMC5928414; doi:10.1126/science.aan1478)
Supplement: Supplementary file 1 [file Science-358-518-s1.pdf]

www.sciencemag.org/content/358/6362/518/suppl/DC1

Supplementary Materials for

Plasmepsins IX and X are essential and druggable mediators of malaria parasite egress and invasion

Armiyaw S. Nasamu, Svetlana Glushakova, Ilaria Russo, Barbara Vaupel, Anna Oksman, Arthur S. Kim, Daved H. Fremont, Niraj Tolia, Josh R. Beck, Marvin J. Meyers, Jacquin C. Niles, Joshua Zimmerberg, Daniel E. Goldberg*

*Corresponding author. Email: dgoldberg@wustl.edu

Published 27 October 2017, *Science* **358**, 518 (2017)
DOI: 10.1126/science.aan1478

This PDF file includes:

Materials and Methods
Figs. S1 to S9
References

Materials and Methods

Reagents and antibodies

All primers were obtained from Integrated DNA Technologies. Restriction enzymes were purchased from New England Biolabs. Anti-HA antibodies were obtained from Sigma while the anti-Flag antibody was from ThermoFisher. Anti-SUB1 and SERA5 antibodies were kind gifts of Dr. Michael J. Blackman (The Francis Crick Institute, London). Monoclonal antibody 2.29 (anti-RAP-1) and monoclonal antibody 12.4 (MSP1) were obtained from The European Malaria Reagent Repository (http://www.malariaresearch.eu). The anti-HAD1 antibody was a kind gift of Dr. Audrey Odom John (Department of Pediatrics, Washington University School of Medicine, St. Louis, Missouri). Anti-Myc antibody (9E10) was purchased from ThermoFisher. aTc was purchased from Cayman Chemical.

Primers

Primers for tagging PMX with 3' Aptamers, 3XHA tag and GFP and for making PMX complementation plasmids

LHR means left homologous region, RHR means right homologous region.

Primers for tagging PMIX with 3' Aptamers and 3XHA

	Sequence	Notes
RHR F	GTACAAACCCGGAATTCGAGCTCGGCAGAG GAAAACGAAAAATATCTCAGTTCCTTACAT	Forward primer for PCR of the RHR of PMIX
RHR R	GGGTATTAGACCTAGGGATAACAGGGTAAT CATTTGATGTTCCACCTTATTGCGAATTGG	Reverse primer for PCR of the RHR of PMIX
INT P1	ATAGGAGTGGAGACATTTAAGATTGGAC	Forward primer for checking integration of donor plasmid at 5' end PMIX genomic locus
INT P2	TCACAGATCTTCTTCGCTGATGAGTTTCT	Reverse primer for checking integration of donor plasmid at 5' end PMIX genomic locus
SgRNA oligo 1	TAAGTATATAATATTCTATGACGTAAGTCAA GTTGGTTTTAGAGCTAGAA	Forward primer for guide RNA used for editing the PMIX locus
SgRNA oligo 2	TTCTAGCTCTAAAACCAACTTGACTTACGTC ATAgAATATTATATACTTA	Reverse primer for guide RNA used for editing the PMIX locus
sgRNA sequence	CTATGACGTAAGTCAAGTTG	Sequence of the guide RNA used for editing the PMIX locus
PM2GT PMIX RHR F	ATACGATTTAGGTGACACTATAGAACTCGA GCAGAGGAAAACGAAAAATATCTCAGT	Forward primer for PCR of RHR for 3XHA PMIX tagging
PM2GT PMIX RHR R	ATCTACCCCTCCAAATATTAATGCAGACTTA AGCATTTGATGTTCCACCTTATTGCGA	Reverse primer for PCR of RHR for 3XHA PMIX tagging

PM2GT PMIX LHR F	ATTCGCAATAAGGTGGAACATCAAATGCTT AAGTCTGCATTAATATTTGGAGGGGTA	Forward primer for PCR of LHR for 3XHA PMIX tagging
PM2GT PMIX LHR R	GTCCGGGACGTCGTACGGGTACCTAGGCAG GTTGTTGATTTTGTTGTGCAG	Reverse primer for PCR LHR for 3XHA PMIX tagging
PM2GT PMIX_P 1	ATAGGAGTGGAGACATTTAAGATTGGAC	Forward primer for checking CRISPR mediated integration of any gene tagged using the PM2GT plasmid
PM2GT_ P2	TATATTATATAACTCGACGCGGCCGTC	Reverse primer for checking CRISPR mediated integration of any gene tagged using the PM2GT plasmid

Primers for tagging PMX with 3' Aptamers, 3XHA and GFP.

Primer name	Sequence	Notes
054_ PMX_ LHR F	GTGCGGCCGCCGGTTTACCGAGCTCTTATTG GTTTTCAAACTTCATTGACTGTGCCGGCCGG CCTC	Forward primer for PCR of the LHR of PMX
054_ PMX_ LHR R	ATCATCATCATCTTTATAATCCGCGATCGCG TTTTTACTTTTTGCTCTTGCTACTCCAAC	Reverse primer for PCR of the LHR of PMX
054_ PMX_ RHR F	AGGTAGCGAATAATACGACTCACTATACTCT TTATTGAATCCCATATTTTAATATACC	Forward primer for PCR of RHR of PMX
054_ PMX_ RHR R	GGGTATTAGACCTAGGGATAACAGGGTAAT TATACTAGTGTATAACCTATTACATCACAT	Reverse primer for PCR of RHR of PMX
LHR seq 1	GACACAGGTACATCTTATAATACAATG	Primer for sequencing LHR
054_ PMX_ LHR_ GFP F	AGAGCAAAAAGTAAAAACGCGATCGCGATG CCTAGGCCAGGTGCAGCACATTATGCAGCA	Forward primer for PCR of GFP to tag the coding sequence of PMX
054_ PMX_ LHR _ GFP R	ATTTATCATCATCATCTTTATAATCTCATTTG TACAGTTCATCCATGCCATGTGTAATCC	Reverse primer for PCR of GFP to tag the coding sequence of PMX
054_ PMX	CTCCTTATGATGGGAAGTCAACTTTA	Forward primer for checking integration of donor plasmid at 5'

INT P1		end of the PMX genomic locus
INT P2	TCACAGATCTTCTTCGCTGATGAGTTTCT	Reverse primer for checking integration of donor plasmid at 5' end of the PMX genomic locus
sgRNA oligo 1	TAAGTATATAATATTATTAATAATGCATTAAAAAAGTTTTAGAGCTAGAA	Forward primer for guide RNA used for editing the PMX locus
sgRNA oligo 1	TTCTAGCTCTAAAACTTTTTTAATGCATTATTAATAATATTATATACTTA	Reverse primer for guide RNA used for editing the PMX locus
sgRNA sequence	ATTAATAATGCATTAAAAAA	Sequence of the guide RNA used for editing the PMX locus
PM2GT PMX RHR F	TACGATTTAGGTGACACTATAGAACTCTCTTTATTGAATCCCATATTTTAATATACCC	Forward primer for PCR of the RHR to tag PMX with 3xHA
PM2GT PMX RHR R	TTCATCTAATTTTACTTCCCAGTAGGACTTAAGCTCGTATACTAGTGTATAACCTATTAC	Reverse primer for PCR of the RHR to tag PMX with 3xHA
PM2GT PMX LHR F	GTAATAGGTTATACACTAGTATACGAGCTTAAGTCCTACTGGGAAGTAAAATTAGAT	Forward primer for PCR of the LHR of PMX to tag PMX with 3xHA
PM2GT PMX LHR R	CGGGACGTCGTACGGGTACCTAGGGTTTTTACTTTTTGCTCTTGCTACTCCAACCATA	Reverse primer for PCR of the LHR of PMX to tag PMX with 3xHA
PMX prom-AatII	GAAAAGTGCCACCTGACGTCGTTAAATAATTATTAATTTTCCATAACCC	Forward primer for PCR of the 5'UTR+ wildtype coding sequence of PMX for complementation
PMX orf rev-NheI	GAAATCAACTTTTGTTCGCTAGCGTTTTTACTTTTTGCTCTTGCTACTCCAACC	Reverse primer for PCR of the 5'UTR+ wildtype coding sequence of PMX for complementation
D266GMUT	CAAACTGTATATCCAATATTTGGTACAGGAAGTACAAATGTATGGGTAGTGACAACA	Primer for site directed mutagenesis to create D266G from the wild-type plasmid

Primers for tagging SUB1 with 3XHA

	Sequence	Notes
SUB1 RHR F	ATACGATTTAGGTGACACTATAGAACTTGGA AATGGCTTTTTAAATAATATATATTTC	Forward primer for PCR of RHR for 3XHA SUB1 tagging
SUB1 RHR R	TCATAAACAGTAGATAAGATAGGCTTAAGAC GTGCTTGCTAATATTAACACACTACA	Reverse primer for PCR of RHR for 3XHA SUB1 tagging
SUB1 LHR F	ATGTGTAGTGTGTTAATATTAGCAAGCACGT CTTAAGCCTATCTTATCTACTGTTTAT	Forward primer for PCR LHR for 3XHA SUB1 tagging
SUB1 LHR R	CCGGGACGTCGTACGGGTACCTAGGATGCAA ATATCTACTTTTTTTTTCCACTTGTA	Reverse primer for PCR of LHR for 3XHA SUB1 tagging
PM2GT_SUB1 P1	GTAGTCATCCTAAATCGTCAACACC	Forward primer for checking CRISPR mediated editing of the SUB1 locus
PM2GT_P2	TATATTATATAACTCGACGCGGCCGTC	Reverse primer for checking CRISPR mediated integration of any gene tagged using the PM2GT plasmid
sgRNA oligo 1	TAAGTATATAATATTGCATTAACTAGTACATC AAAGTTTTAGAGCTAGAA	Forward primer for guide RNA used for editing the SUB1 locus
sgRNA oligo 2	TTCTAGCTCTAAAACTTTGATGTACTAGTTAA TGCAATATTATATACTTA	Reverse primer for guide RNA used for editing the SUB1 locus
sgRNA sequence	GCATTAACTAGTACATCAAA	Sequence of the guide RNA used for editing the SUB1 locus

Primers for tagging RON4 with 3XHA

	Sequence	Notes
RON4 RHR F	cgatttaggtgacactatagaactTCTGTTTATACAAGTATAAGCCCAAACAAACAT	Forward primer for PCR of RHR for 3XHA RON4 tagging
RON4 RHR R	CTGAACATTTATATAACATATCTGTTAGGCGATCGCATAAGAATGTATAGGAATATGTTC	Reverse primer for PCR of RHR for 3XHA RON4 tagging
RON4 LHR F	GAACATATTCCTATACATTCTTATGCGATCGCCTAACAGATATGTTATATAAATGTTCAG	Forward primer for PCR of RHR for 3XHA RON4 tagging
RON4 LHR R	GCGTAGTCCGGGACGTCGTACGGGTATAAATCATCAAAAATCATCTTTTCAGCATCGTCT	Reverse primer for PCR of LHR for 3XHA RON4 tagging
PM2GT_RON4 P1	AGGCAACAGCTCAAAGAATTAATTCTTAC	Forward primer for checking CRISPR mediated editing of the RON4 locus
PM2GT_P2	TATATTATATAACTCGACGCGGCCGTC	Reverse primer for checking CRISPR mediated integration of any gene tagged using the PM2GT plasmid
sgRNA oligo 1	TATTAAGTATATAATATTTCTGTTTATACAAGTATAAGGTTTTAGAGCTAGAAATAG	Forward primer for guide RNA used for editing the RON4 locus
sgRNA oligo 2	CTATTTCTAGCTCTAAAACCTTATACTTGTATAAACAGAAATATTATATACTTAATA	Reverse primer for guide RNA used for editing the RON4 locus
sgRNA sequence	TCTGTTTATACAAGTATAAG	Sequence of the guide RNA used for editing the RON4 locus

Plasmid Cloning

All plasmids were verified by sequencing.

Plasmid cloning for aptamer regulation of PMIX

To install aptamers on the 3' end of PMIX, the pMG57 plasmid (*12*) was modified as follows. Briefly, the TetR-DOZI$_{2A}$BSD$_{2A}$RLUC cassette was moved from pMG57 into the NotI site of the linear pJAZZ® plasmid (Lucigen). A multiple cloning site was also added to the plasmid allowing the next cloning steps. The linear vector was then modified to install the 10X aptamer and HSP86 3'UTR. Upstream of the 10X aptamers, an AsiSI site was inserted to allow cloning of LHRs. Downstream of the TetR-DOZI-BSD-RLUC cassette, an I-SceI site was inserted to clone RHRs (see Fig. S1 for illustration of this modified vector).

The RHR (RHR), ~450bp of PMIX coding sequence immediately downstream of the stop codon of PMIX, was PCR amplified and cloned into the I-SceI site of the parent plasmid described previously. The 3' region of the coding sequence of PMIX has short exons separated by introns. To allow efficient cloning and sequencing, the last 3 introns were removed and the exons were combined to make a contiguous coding sequence. These exons were re-codonized to *Toxoplasma gondii* codon bias. The exon before the most upstream intron was used as the LHR (LHR). Together, the LHR and re-codonized portion were ordered as a gene block from IDT. This gene block was cloned, using Gibson Assembly®, into AsiSI sites of the RHR containing plasmid, creating an in-frame fusion with a 2xFLAG tag at the 3' end of the LHR. Below is the sequence of the LHR (bold) and the re-codonized region (introns removed- italics). Gibson cloning overhangs are in lowercase. FLAG tags are underlined.

ggttttcaaacttcattgactgtgccggccggcc**TCTGCATTAATATTTGGAGGGGTAGATAAAAATTT TTTTGAAGGAGATATATATATGTTTCCTGTTGTTAAGGAATATTATTGGGAAAT CCATTTTGATGGTTTATATATTGATCATCAGAAATTTTGTTGTGGTGTTAATTCT ATTGTATATGATTTAAAAAAAAAAAGATCAAGAAAATAATAAATTATTTTTTACGA GAAAATATTTTAGAAAAAATAAATTCAAAACTCATTTAAGAAAATATCTTCTTAA AAAAATAAAACATCAAAAAAAAACAAAAACATTCTAATCATAAAAAAAAAAAATT AAACAAGAAAAAAAATTACTTAATTTTTGATTCTGGAACATCTTTTAATAGTGTC CCAAAGGATGAAATCGAATATTTCTTTCGTGTCGTTCCTTCAAAG***AAGTGCGACG ACTCGAATATTGACCAGGTTGTCTCGAGTTATCCCAACCTCACTTACGTGATCAATAAGATG CCGTTTACATTGACACCTAGCCAATACCTGGTTAGGAAAAATGACATGTGCAAACCTGCTT TCATGGAGATTGAAGTTTCCAGTGAGTACGGGCACGCGTACATCTTGGGAAACGCCACCT TCATGAGGTATTACTACACTGTGTACCGTCGAGGAAACAATAATAACTCTTCTTACGTTGGA ATCGCTAAAGCAGTCCACACCGAGGAGAATGAAAAATATCTCTCGTCCCTGCACAACAAAA TCAACAACCTG*GACTACAAGGACGACGATGACAAGgcgatcgcggattataaagatgatgatgataaatga cgtacgg

Plasmid cloning for aptamer regulation of PMX

The RHR of PMX (400bp of 3'UTR) was cloned into the I-SceI site of the parent plasmid described previously. The resulting plasmid was digested with AsiSI and the LHR PCR product (432bp upstream of the stop codon) was cloned into this site. The PMX gene was tagged with a 1X FLAG tag.

PMIX plasmid for creating the PMIX and X double aptamer regulation strain
To create the PMIX and X dual aptamer regulated line (Fig. S2), the PMIX aptamer plasmid described previously was modified by replacing the TetR-DOZI-BSD cassette with a yDHODH resistance gene cassette (Fig. S2) using the BmtI and AhdI restriction sites. The yDHODH cassette was PCR amplified from the pyEOE plasmid (*23*).

Cloning of plasmids for tagging PMIX, PMX, SUB1 and RON4 with a triple Hemagglutinin (3X HA) tag
For tagging genes with a 3XHA, the PM2GT plasmid (*24*) was used. This plasmid was modified to replace the GFP gene with a 3XHA tag (TACCCGTACGACGTCCCGGACTACGCTGGCTATCCCTATGATGTGCCCGATTATGCG TATCCTTACGATGTTCCAGATTATGCC) using the QuickChange Lightning Multi-Site mutagenesis kit (Agilent). The modified PMGT_3XHA plasmid was digested with AvrII and XhoI. The RHR and LHR pieces of the genes of interest were PCR amplified from NF54attB genomic DNA and attached to each other via Gibson assembly. The RHR+LHR combination was then PCR amplified using the RHR forward primer and LHR reverse primer and this PCR product was cloned into the linearized PM2GT plasmid. An AflII site was inserted between the RHR and LHR pieces. After cloning the piece into PM2GT_3XHA plasmid, the plasmid was linearized with AflII for transfection. Because the LHR of RON4 contained an AflII site, an AsiSI site was inserted between the RHR and LHR for linearization pre-transfection.

Cloning of sgRNAs for CRISPR/Cas9 modification of gene loci

Complementary sense and antisense oligos encoding the sgRNAs for CRISPR/Cas9 editing were annealed and cloned into the U6 promoter-based sgRNA expression cassette of the pyAIO plasmid as previously described (*25*).

Cloning of complementation plasmids

To complement the PMX knockdown, we first modified the plasmid pyEOE (*24*) to allow for Bxb1 integrase-mediate genomic integration (*27*) by inserting a 2xattP sequence into the vector backbone sequence using the QuickChange Lightning Multi-Site mutagenesis kit (Agilent) with the primer 5'-
GGTCGACTCTAGAGGATCCCCGGGTACCGAGCTCGAATTCTGGTTTGTCTGGTCAAC CACCGCGGTCTCAGTGGTGTACGGTACAAACCCGAATTCTGGTTTGTCTGGTCAACC ACCGCGGTCTCAGTGGTGTACGGTACAAACCCGGAATTCTAGATTTAATAAATATGT TCTTATATATAATG -3'. A 3xMYC epitope tag was then inserted between XhoI and EagI using the QuickChange Lightning Multi-Site mutagenesis kit and the primer 5'-
CGAATAAACACGATTTTTTCTCGAGGCTAGCGAACAAAAGTTGATTTCTGAAGAAGA TTTGAACGGTGAACAAAAGCTAATCTCCGAGGAAGACTTGAACGGTGCTAGGGCCG AGGAGCAGAAGCTGATCTCCGAGGAGGACCTGTGACGGCCGCGTCGAGTTAT-3'. An NheI site was included at the 5' end of the 3xMYC sequence. This resulted in the vector pyEOE-

2xattP-3xMYC. The PMX genomic locus including ~2kb upstream of the start codon (promoter region) and the entire coding sequence was PCR amplified from NF54 genomic DNA (~3.7kb total). This PCR product was cloned into the pyEOE-2xattP-3xMYC plasmid between the XhoI and NheI sites, resulting in the plasmid pyEOE-2xattP-PMX-3xMYC-WT. This plasmid was then mutagenized (QuikChange®, Agilent) to create the D266G mutant using primers listed previously.

Cloning PMX and SUB1 for expression in mammalian cells

To express PMX in mammalian cells, an *S. cerevisiae* recodonized PMX gene (a truncated version comprising ~40kDa of the catalytic domain) was cloned into the AgeI and KpnI sites of the vector pHLSEC (Addgene) by Gibson assembly. The recodonized sequence is shown here.

ATGTCAGATAATTCCAGTATCGAGAAAAACTTTATCGCTCTTGAAAATAAGAACGCT
ACCGTGGAGCAGACCAAAGAGAATATCTTCCTGGTGCCATTGAAGCATCTAAGGGA
CTCACAATTTGTCGGAGAATTGTTGGTAGGCACACCCCCACAGACTGTGTATCCAAT
CTTCGACACCGGCTCTACTAACGTTTGGGTAGTCACTACTGCCTGTGAGGAGGAAAG
TTGTAAAAAAGTGCGTAGGTACGATCCCAACAAATCCAAAACATTCAGGAGGTCCT
TTATTGAAAAGAATTTACATATTGTATTTGGGTCTGGATCTATAAGTGGTTCCGTCGG
TACCGATACCTTTATGTTGGGGAAACATCTGGTCAGAAACCAAACATTTGGCTTAGT
TGAATCCGAGAGCAATAATAACAAGAATGGTGGAGATAATATTTTCGACTATATATC
TTTTGAAGGAATTGTTGGCCTGGGCTTTCCAGGCATGTTAAGTGCAGGTAACATTCC
TTTTTTCGATAACCTTTTAAAGCAAAATCCAAACGTCGATCCCCAATTTTCTTTCTAT
ATATCTCCTTACGATGGCAAAAGCACTCTAATCATCGGTGGCATCTCTAAATCTTTCT
ACGAGGGAGATATCTATATGCTTCCTGTCCTGAAGGAATCCTATTGGGAAGTTAAGC
TGGACGAACTATACATCGGAAAAGAGCGTATATGCTGCGATGAGGAAAGTTATGTG
ATATTTGACACGGGGACATCATACAATACAATGCCTTCCTCTCAGATGAAGACATTT
CTGAATCTAATTCACAGCACGGCGTGCACCGAGCAAAATTATAAAGACATATTGAA
GTCCTACCCAATTATCAAATACGTCTTTGGAGAACTGATCATCGAATTACACCCGGA
AGAATATATGATATTAAATGATGACGTATGCATGCCTGCTTACATGCAGATAGACGT
CCCCTCTGAGAGGAACCATGCCTACCTGCTTGGTTCTCTGAGCTTTATGCGTAACTTT
TTCACGGTCTTTGTCAGGGGGACTGAAAGCAGGCCAAGCATGGTCGGTGTCGCACGT
GCTAAAAGCAAGAACTATCCTTACGATGTCCCGGACTACGCTTACCCGTATGATGTG
CCTGACTACGCATATCCGTACGACGTTCCTGACTACGCG

To express SUB1 p82 in mammalian cells, a mammalian re-codonized SUB1 gene (full-length minus the signal peptide) was cloned into the AgeI and KpnI sites of pHLSEC. The recodonized sequence is shown below.

AAGGAAGTGAGGTCCGAGGAAAACGGTAAAATCCAGGACGACGCGAAAAAAATTG
TGTCTGAATTGCGCTTCCTTGAAAAGGTTGAGGATGTTATTGAAAAGTCTAACATTG
GCGGGAACGAGGTAGATGCGGACGAGAACTCCTTTAACCCTGATACGGAAGTCCCC
ATCGAAGAGATTGAGGAGATAAAAATGCGCGAACTGAAAGATGTGAAAGAAGAAA
AGAATAAAAATGACAATCATAACAATAATAACAACAATATAAGTTCCAGCAGCTCA
AGCTCCTCAAACACGTTTGGGGAAGAGAAGGAGGAGGTTTCAAAGAAGAAAAAGA

AGCTGCGCCTTATAGTTAGCGAAAATCACGCAACCACACCAAGTTTTTTCAGGAAT
CTCTGCTGGAACCGGACGTATTGTCATTTCTCGAGTCTAAGGGCAATCTTAGCAACC
TCAAGAATATCAATTCTATGATCATAGAGCTCAAAGAGGACACGACCGATGATGAG
CTTATTAGCTATATCAAAATTCTTGAGGAAAAGGGTGCCCTTATTGAATCCGATAAA
CTCGTCTCAGCCGATAACATTGATATCAGCGGGATAAAGGATGCAATCCGGAGAGG
AGAAGAGAACATTGATGTCAATGATTACAAATCTATGTTGGAAGTAGAAAACGACG
CAGAGGATTATGACAAGATGTTTGGTATGTTTAATGAAAGTCATGCCGCGACTAGTA
AACGAAAAAGGCATTCCACCAATGAAAGAGGATACGACACATTCAGTAGTCCATCA
TACAAGACTTACTCCAAAAGTGATTACTTGTACGACGATGACAATAACAACAATAAT
TATTATTATTCTCACTCATCTAACGGACACAACAGCAGTTCCAGAAACTCATCCTCTT
CCCGGAGTCGGCCGGGCAAGTATCATTTCAACGACGAGTTCCGAAATCTGCAGTGG
GGATTGGATCTCTCCCGGCTTGACGAAACCCAAGAACTGATAAATGAGCACCAGGT
GATGAGCACTAGAATCTGCGTGATAGACAGCGGTATTGACTACAATCATCCAGATCT
GAAGGATAATATAGAATTGAATCTCAAGGAGCTCCATGGGCGCAAAGGCTTTGATG
ATGATAACAACGGGATTGTCGATGACATTTATGGAGCCAACTTTGTGAATAACTCCG
GAAATCCTATGGATGATAATTACCACGGAACGCACGTATCCGGCATCATAAGCGCC
ATCGGAAACAACAACATCGGGGTAGTCGGAGTTGACGTGAATAGTAAGCTGATCAT
TTGCAAAGCCTTGGACGAGCATAAGCTTGGCCGACTTGGCGACATGTTCAAATGCTT
GGACTATTGCATATCCCGCAATGCGCATATGATCAATGGCTCATTCTCTTTTGACGA
ATACTCAGGGATATTTAATTCCTCCGTTGAGTATTTGCAAAGAAAGGGTATTCTCTTT
TTTGTATCCGCATCAAATTGTAGCCATCCCAAATCTAGTACGCCCGATATCCGCAAG
TGCGATCTGTCCATAAATGCCAAATACCCGCCGATATTGAGCACTGTCTATGATAAC
GTAATATCCGTAGCTAACCTGAAAAAAAATGATAACAACAACCACTACAGCTTGTCT
ATTAATTCATTCTACAGCAACAAGTACTGCCAGCTCGCTGCCCCGGGTACAAATATA
TATAGCACGGCGCCCCACAATAGTTACCGGAAGCTTAATGGGACATCCATGGCCGC
ACCGCATGTCGCGGCCATCGCATCCCTCATATTCTCTATCAACCCCGACCTCTCTTAC
AAAAAGGTTATCCAAATACTTAAAGATAGTATAGTATATTTGCCAAGCCTCAAGAAC
ATGGTTGCATGGGCCGGTTATGCCGACATTAACAAGGCCGTAAACTTGGCAATCAA
GTCAAAGAAGACTTATATTAATAGCAATATATCTAACAAATGGAAAAAGAAGAGTA
GGTACCTCCAT

Parasite culture, transfection and synchronization

NF54attB parasites (*28*) and resultant transgenic strains were cultured in human red blood cells as previously described (*26*). For creating the aptamer-regulated PMIX and X lines, 50µg of aptamer plasmid was co-transfected with 50µg of the sgRNA and Cas9-carrying plasmid. Immediately following transfection, 1µM aTc was introduced into the cultures and maintained from that time forward. Parasites were selected with 2.5ug/ml BSD beginning 24 hours after transfection.

For complementation of PMX, plasmids carrying WT or mutant PMX coding sequences were independently co-transfected with the Bxb1 integrase (*27*) plasmid into the PMX^{apt} parasites. Parasites were selected with 12.5µg/ml DSM-1 together with 2.5µg/ml BSD and 1µM aTc. The integration of these plasmids into PMX^{apt} strain was confirmed by PCR using the following

primers: 5'-TTTTTGTAGACCCCATTGTGAGTACATAAAT-3' and 5'-CATTTGAATTATTGCTCAACGCT-3'

Highly synchronous ring-stage parasites were obtained as follows. High parasitemia schizonts cultures were passed through MACS LD magnet columns (Miltenyi Biotec) and schizonts were collected. These were then added to fresh uninfected RBCs resuspended in warm culture media. The cultures were shaken at 80 RPM for 2 hours and the resulting parasites were synchronized using 5% sorbitol and returned into culture.

For analysis of PMX^{apt} parasite analysis, the population of parasites generated was cloned. The five clones picked behaved the same in growth assays and two clones were used for the rest of our experiments.

Western blotting analyses

For PMIX and X knockdown western blots (Fig. 1A), highly synchronous ring-stage parasites were washed three times (10 minute wait each time), supplemented with fresh blood and split into +/- aTc plates. After 24 hours the cultures were treated with 500 nM compound 1 (*29*) to prevent egress, allowing the accumulation of parasites as late, segmented schizonts. The schizonts were collected using MACS columns, lysed in RIPA buffer supplemented with protease inhibitor cocktail, sonicated for 15 seconds and centrifuged 3x at 13300g for 10 minutes each time. Supernatants were transferred to new tubes after each spin to get rid of hemozoin. Supernatants were mixed with 5X sample buffer and boiled at 99°C for 5 minutes.

For MSP1 western blotting (Fig. 3D), highly synchronous ring-stage parasites were washed three times (10 minute wait each time), supplemented with fresh blood and split into +/- aTc plates. After 24 hours the cultures were treated with 500 nM compound 1 (*29*) to prevent egress, allowing the accumulation of parasites as late, segmented schizonts. After 50 hours, the schizonts were washed 3x to get rid of compound 1. One hour later the schizonts were collected using MACS columns and processed similarly to what was done for Fig. 1A.

For all other western blots, highly synchronous (2-hour window) parasites were created. These were washed to eliminate aTc as described before and split into +/- aTc conditions. The parasites were carefully monitored until new rings started emerging, suggesting that parasites had reached the end of their cycle. The schizonts in the cultures were then purified by MACS columns and processed for western blotting as described previously.

For western blots showing effects of PMX inhibitor treatment (Fig. 4E, F), compounds were added to trophozoites and schizonts were harvested and processed for western blotting.

Total protein was separated by SDS-PAGE. The resolved proteins were transferred onto a nitrocellulose membrane (Bio-Rad). The membrane was then blocked with blocking buffer (Licor) for 30 minutes and probed with the appropriate primary antibody in Licor blocking buffer and then the appropriate secondary antibody. Blots were visualized on an Odyssey imaging system (Licor). Quantification of band intensity was performed using Image Studio (Licor). All primary antibodies for western blotting were used at 1:1000 dilutions in Licor blocking buffer. The secondary antibodies were used at 1:5000 dilution in PBS+0.05% Tween® 20 (Sigma).

Brefeldin A Experiment

Relatively synchronous troph and schizonts stage NF54attB_PMX_3XHA parasites were harvested by MACs purification. These were split into two cultures, one treated with DMSO vehicle only and the other supplemented with 0.5ug/ml brefeldin A (BFA) (*24)*.
This is 1/10 of the amount of BFA normally used. 4 hours after treatment, parasites were collected and prepared for western blotting for both PMX_3xHA and SUB1.

Electron Microscopy

Infected RBCs were fixed in 4% paraformaldehyde (Polysciences Inc., Warrington, PA) in 100mM PIPES/0.5mM $MgCl_2$, pH 7.2 for 1 hr at 4°C. Samples were then embedded in 10% gelatin and infiltrated overnight with 2.3M sucrose/20% polyvinyl pyrrolidone in PIPES/$MgCl_2$ at 4°C. Samples were trimmed, frozen in liquid nitrogen, and sectioned with a Leica Ultracut UCT cryo-ultramicrotome (Leica Microsystems Inc., Bannockburn, IL). 50 nm sections were blocked with 5% FBS/5% NGS for 30 min and subsequently incubated with appropriate primary antibodies for 1 hr, followed by appropriate secondary antibodies conjugated to 12nm or 18 nm colloidal gold (1:30) for 1 hr. Sections were washed in PIPES buffer followed by a water rinse, and stained with 0.3% uranyl acetate/2% methyl cellulose and viewed on a JEOL 1200EX transmission electron microscope (JEOL USA, Peabody, MA) equipped with an AMT 8 megapixel digital camera (Advanced Microscopy Techniques, Woburn, MA). All labeling experiments were conducted in parallel with controls omitting the primary antibody which was consistently negative at the concentration of colloidal gold conjugated secondary antibodies used in these studies.
For EM without immunostaining, cells were fixed in 2% paraformaldehyde/2.5% glutaraldehyde (Polysciences Inc., Warrington, PA) in 100 mM sodium cacodylate buffer, pH 7.2 for 1 hr at room temperature. Samples were washed in sodium cacodylate buffer and postfixed in 1% osmium tetroxide (Polysciences Inc.) for 1 hr. Samples were then rinsed extensively in dH_20 prior to en bloc staining with 1% aqueous uranyl acetate (Ted Pella Inc., Redding, CA) for 1 hr. Following several rinses in dH_20, samples were dehydrated in a graded series of ethanol and embedded in Eponate 12 resin (Ted Pella Inc.). Sections of 95 nm were cut with a Leica Ultracut UCT ultramicrotome (Leica Microsystems Inc., Bannockburn, IL), stained with uranyl acetate and lead citrate, and viewed on a JEOL 1200 EX transmission electron microscope (JEOL USA Inc., Peabody, MA) equipped with an AMT 8 megapixel digital camera and AMT Image Capture Engine V602 software (Advanced Microscopy Techniques, Woburn, MA).

Flow cytometry

Flow cytometry for determination of parasitemia and progression of the cell cycle was performed by incubating 15 μl of parasites cultures in 165 μl of 0.8 μg ml^{-1} acridine orange in PBS for 1 minute. Stained parasites were analyzed on a BD FACS Canto flow cytometer gating on DNA

and RNA-bound dye signal using FITC and PerCP-Cy5.5 filters, respectively. 50,000 events were recorded for each sample.

Inhibitor Sensitivity Assays

To determine if the inhibitors acted in a PMIX or X dependent fashion, we first performed an aTc titration on our $PMIX^{apt}$ and X^{apt} strains to determine a concentration at which there was a substantial protein knockdown but normal parasite proliferation. At 3nM aTc, parasites consistently expanded similarly to the 10nM aTc condition (Fig. S6) for both strains. To determine shifts in the EC_{50} of these compounds upon KD of PMIX and X, we performed drug assays comparing EC_{50} at 10nM and 3nM aTc. Ring-stage parasites were washed 3X (10 min wait each time) in RPMI medium to remove aTc. The cultures for each were then expanded to larger volumes and split into 10nM and 3nM aTc conditions. These were plated in 24-well plates with triplicates for each inhibitor concentration. These experiments were repeated at least 3 times to evaluate shifts in dose response. The inhibitors were dissolved in 100% DMSO. The final DMSO concentration in these assays was 0.1%.

Live cell imaging

We performed live-cell recording as described (13) for assessment of parasite egress with some modifications to evaluate parasite invasion as well. Briefly, erythrocytes infected with late-stage parasites were isolated and mixed with uninfected erythrocytes to 1% hematocrit and placed in environmental chambers for two hours. Three hundred late stage parasites were monitored for egress in each experiment. Parasites were scored as egressed or not. Additionally, for parasites that did egress, the number of new invasions per each egress site was scored. Invasion counts were performed for thirty sites of egress in each experiment.

PMX expression and enzyme assays

To produce recombinant PMX (catalytic domain), pHLSEC_PMX was expressed in Expi293 cells (Thermo Fisher). Briefly, cells were seeded at 5 x 10^6 cells/ml a day prior to transfection. 200 μg of pHLSEC-PMX was diluted in Opti-MEM (Thermo Fisher) and incubated with HYPE-5 transfection reagent (Oz Biosciences). The DNA and transfection reagent complex was then added dropwise to cells. Transfected cells were supplemented daily with Expi293 expression medium (Thermo Fisher) and 2% (w/v) Hyclone Cell Boost Supplement (GE). The growth medium was collected after 4 days and 0.22μm filtered (Corning). The supernatant was concentrated using 10 kDa MWCO concentrators (Amicon® Ultra-15). Reactions (200μl) contained 50mM NaOAc/20mM NaCl pH 5.0 with 5μl of concentrated supernatant (enzyme), 0.5μM of the fluorogenic aspartic protease substrate M-2455 (Bachem) and various dilutions of inhibitor. Fluorescence was measured every two minutes over a two-hour time course using a Cytation™ 3 (BioTek). Protein prepared from a no PMX control plasmid was used as control. Pepstatin was used as an aspartic protease inhibitor control. DMSO solvent was kept at 0.1% for all samples.

SUB1 expression and cleavage

Full-length SUB1 was expressed in Expi293 cells as for PMX above. SUB1 was incubated with PMX for 4 hrs at 37 °C in assay buffer described in the previous section. SUB1 cleavage was assessed by western blot as above.

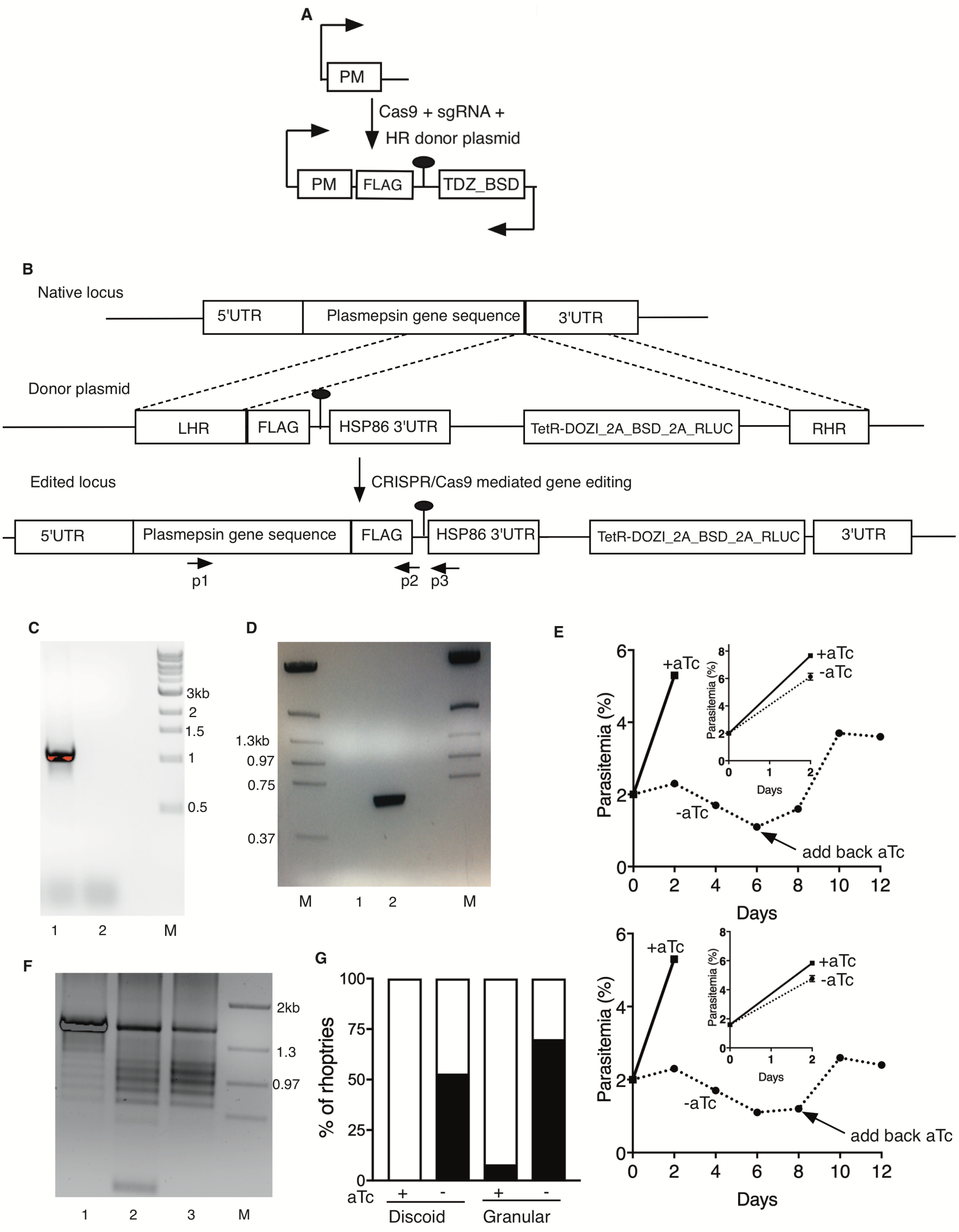
A
PM
Cas9 + sgRNA +
HR donor plasmid
PM
FLAG
TDZ_BSD
B
Native locus
5'UTR
Plasmepsin gene sequence
3'UTR
Donor plasmid
LHR
FLAG
HSP86 3'UTR
TetR-DOZI_2A_BSD_2A_RLUC
RHR
Edited locus
CRISPR/Cas9 mediated gene editing
5'UTR
Plasmepsin gene sequence
FLAG
HSP86 3'UTR
TetR-DOZI_2A_BSD_2A_RLUC
3'UTR
p1
p2
p3
C
3kb
2
1.5
1
0.5
1
2
M
D
1.3kb
0.97
0.75
0.37
M
1
2
M
E
Parasitemia (%)
+aTc
-aTc
add back aTc
Days
Parasitemia (%)
+aTc
-aTc
Days
F
2kb
1.3
0.97
1
2
3
M
G
% of rhoptries
100
75
50
25
0
aTc
+
-
+
-
Discoid
Granular

Fig. S1. Editing of PMIX and X loci to generate aptamer-regulated strains. (**A**) Schematic for modifying PMIX/X loci with TetR-aptamer system. FLAG tag and aptamers (lollipop) were integrated by homologous recombination to repair CRISPR/Cas9-induced double-strand breaks. Upon aTc withdrawal, translation is blocked, leading to protein knockdown (KD). TetR_BSD= TetR/ blasticidin-S deaminase cassette. (**B**) Schematic of the native loci and editing plasmids. 5'UTR= 5' untranslated region; 3'UTR = 3' untranslated region; LHR= LHR; FLAG= flag epitope tag; HSP86 3'UTR= 3' untranslated region of the HSP86 gene used to drive expression of modified plasmepsin gene; TetR-DOZI= Tet repressor protein fused to DOZI effector; 2A= T2A viral skip peptide; BSD= blasticidin resistance marker; RLUC= renilla luciferase; RHR= RHR; p1= PCR primer on genomic locus upstream of LHR; p2= PCR primer on donor plasmid between FLAG and the aptamers (this is a common primer for both PMIX and X). (**C**) PCR diagnostic test for integration in $PMIX^{apt}$ strain using p1 of PMIX and p2. Expected size of PCR product is 1083bp. 1= $PMIX^{apt}$ strain; 2= parental strain; M= DNA ladder (**D**) PCR diagnostic test for integration in PMX^{apt} strain using PMX p1 and p2. Expected size of PCR product is 593bp. 1= parental strain; 2= PMX^{apt} . (**E, F**) Parasites grown in –aTc culture for 3 or 4 cycles become insensitive to aTc withdrawal after they recover. (**E**) Representative growth curves for PMX^{apt} parasites grown in -aTc over 3 or 4 cycles. Synchronous ring stage parasites were grown -aTc and the parasitemia was determined by flow cytometry. After 3 (top) or 4 (bottom) cycles aTc was added back to these cultures and the parasitemia was monitored for 3 more cycles. After the third cycle, recovered parasites were washed 3 times to get rid of aTc and then grown +/-aTc for one more cycle (Inserts in E). Parasitemia was determined by flow cytometry. (**F**) PCR diagnostic test using p1 and p3 to assess the editing and aptamer status of parasites recovered after 3 or 4 cycles of aTc withdrawal. Parasites from day 12 of (E) were collected. Expected size of PCR product is 1.5kb. 1= parental strain; 2= PMX^{apt}; 3=PMX^{apt} recovered after being grown –aTc for 3 cycles; PMX^{apt} recovered after being grown –aTc for 4 cycles. An accumulation of truncated aptamer-array containing parasites was seen over time in culture. (**G**) $PMIX^{apt}$ rhoptry morphology statistics. Rhoptries from 30 cryoEM sections including the ones in Fig.2F-H were scored as bulbar (elongated) or round. Of round cross-sections, 0/26 displayed discoid morphology in the +aTc culture and 25/47 in the –aTc culture. Of bulbar cross-sections, 2/26 displayed granular morphology in the +aTc culture and 16/23 in the –aTc culture. ****p<.0001 for each (Fisher's exact test).

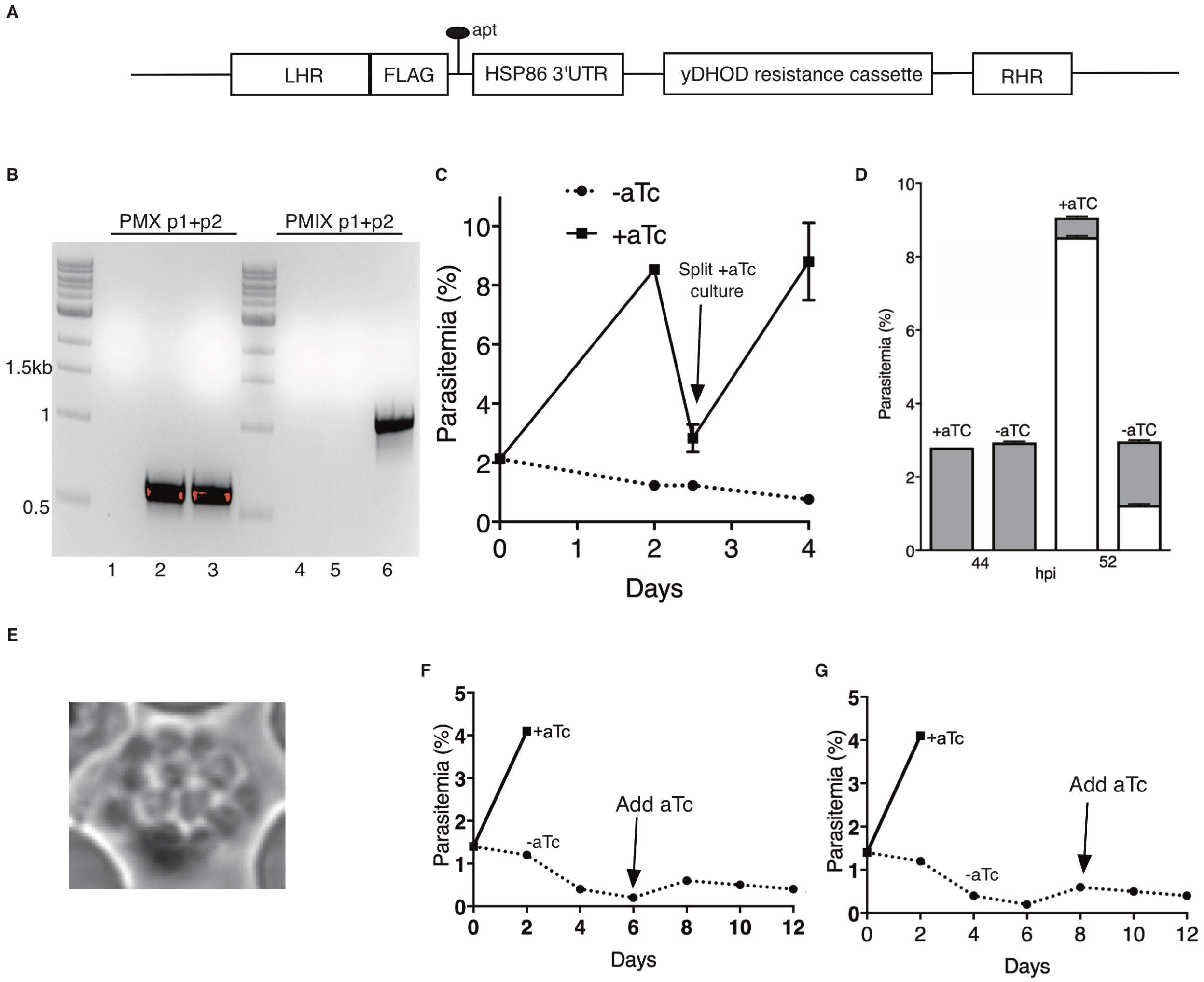

Fig. S2. Editing the PMX^{apt} strain with a PMIX donor vector to create the PMIX^{apt}/X^{apt} double knockdown strain. (**A**) Schematic of the PMIX plasmid modified from the one in Fig. S1 by replacement of the TetR_BSD_RLUC region with yDHODH resistance marker. All labels are the same as in Fig. S1. (**B**) PCR diagnostic test of PMIX^{apt}/X^{apt} to confirm editing. 1, 4= parental strain; 2, 5= PMX^{apt} strain; 3, 6= PMIX^{apt}/X^{apt} strain. Expected PCR product sizes are 593 bp and 1083 bp for PMX and PMIX editing respectively. (**C**) Representative growth curve for PMIX^{apt}/X^{apt} parasites over two cycles. Synchronous ring stage parasites were grown +/-aTc and the parasitemia was determined by flow cytometry. The +aTc culture was split back to ~2% parasitemia and supplemented with fresh RBCs on day 2 (arrow) to avoid overexpansion (**D**) Parasite stage upon maturation ± aTc. Parasites from (C) were monitored at 44 and 52 hpi. Schizonts and new rings were counted by flow cytometry. (E) Merozoite cluster typical of culture –aTc. (**F,G**) PMIX^{apt}/X^{apt} grown in –aTc media for 3 cycles (F) or 4 cycles (G) do not reappear when supplemented with aTc again.

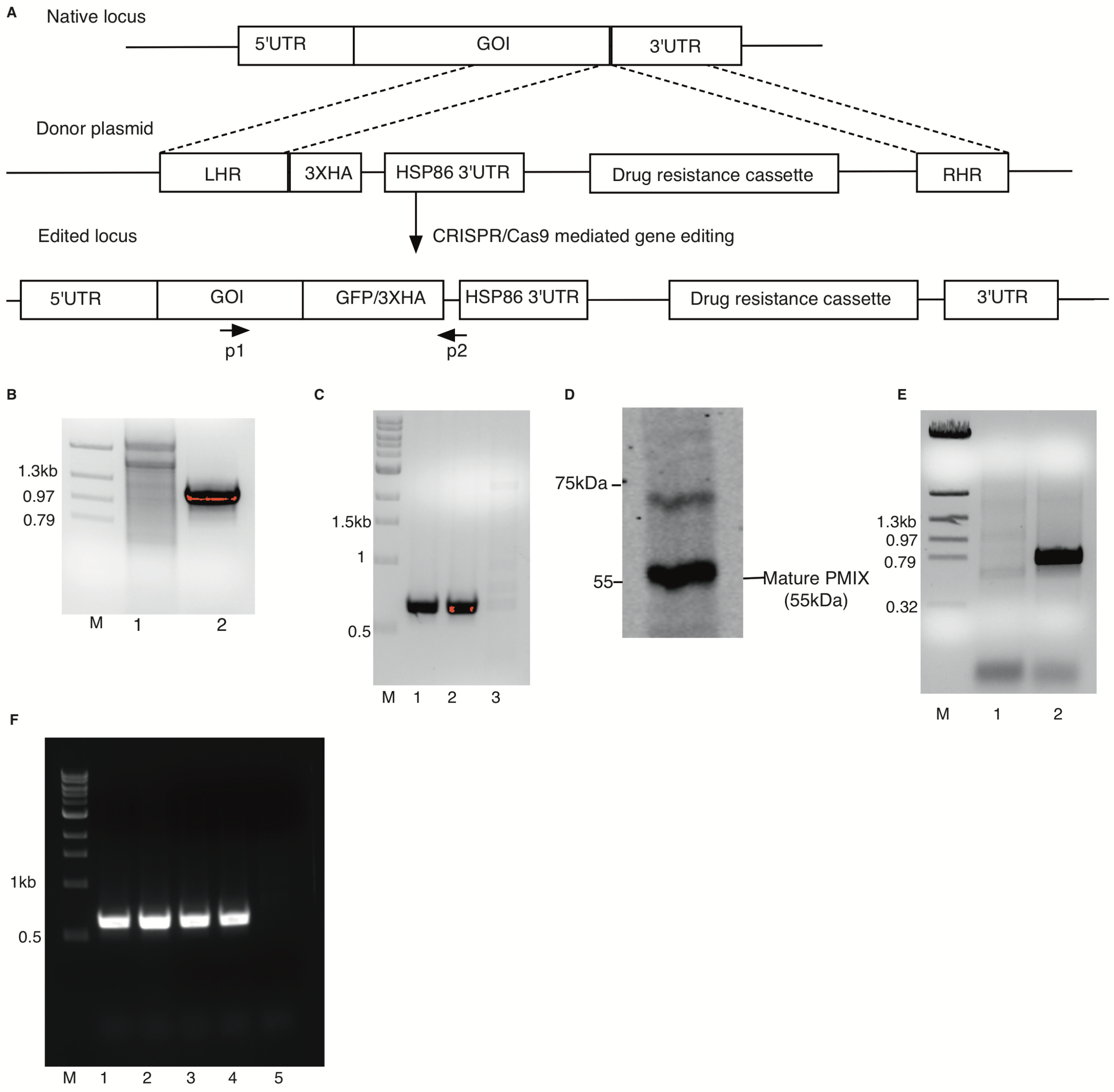

Fig. S3. Editing of PMIX, PMX, RON4 and SUB1 loci to generate triple HA (3X HA) and GFP tagged genes. (**A**) Schematic of the native loci and editing plasmids. 5'UTR= 5' untranslated region; 3'UTR = 3' untranslated region; LHR= LHR; HA= hemagglutinin epitope tag; GFP= green fluorescent protein; HSP86 3'UTR= 3' untranslated region of the HSP86 gene used to drive expression of the modified plasmepsin gene; yDHODH= yeast dihydroorotate dehydrogenase resistance gene; p1= PCR primer on genomic locus upstream of LHR; p2= PCR primer on donor plasmid downstream of the HA or GFP tags. (**B**) PCR diagnostic test of PMIX_3XHA strain using p1 of PMIX and p2. Expected size of PCR product is 1083bp. (**C**) PCR diagnostic test of PMX_3XHA strain using PMX p1 and p2. Expected size of PCR product is 593bp. 1,2 = PMX_3XHA transfections 1 and 2; 3= parental strain (**D**) Immunoblotting with anti-HA antibody showing correct tagging of PMIX protein. (Correct tagging of PMX is shown in Fig. 3B). (E) PCR diagnostic test of PMIX:RON4_3XHA strain using RON4 p1 and p2.

Expected size of PCR product is ~730bp. 1= PMIX parental strain. 2=PMIX:RON4_3XHA strain. (F) PCR diagnostic test of PMIX:SUB1_3XHA, PMX_SUB1_3XHA and NF54_SUB1_3XHA strains using SUB1 p1 and p2. Expected size of PCR product is ~550bp. 1,2= PMX:SUB1_3XHA; 3= PMIX_SUB1_3XHA, 4= NF54_SUB1_3XHA; NF54 parental strain.

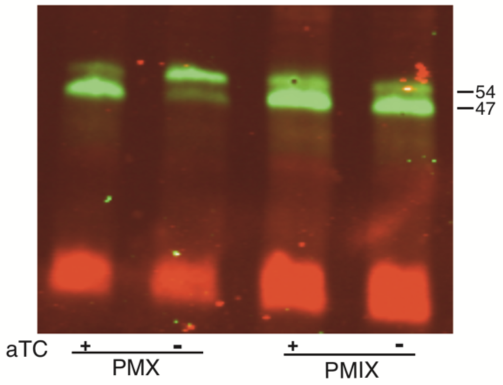

Fig. S4. Knockdown of PMX but not PMIX impairs SUB1 processing. $PMIX^{apt}$ and PMX^{apt} parasites, in which the SUB1 gene was tagged with a 3XHA, were cultured for 46 hpi ±aTc and processed for immunoblot using anti-HA antibodies (green). HAD1 is the loading control (red).

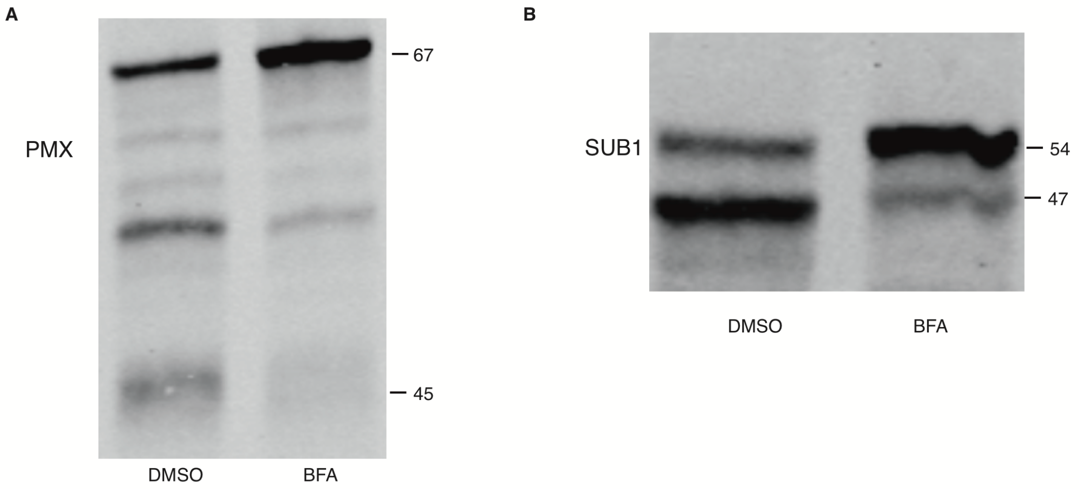

Fig. S5. PMX maturation is blocked by brefeldin A (BFA). (**A, B**) Troph and schizont stage PMX_3XHA parasites were cultured for 4 hours in 0.5ug/ml of BFA and processed for immunoblot using anti-HA antibodies for PMX (A) or anti-SUB1 antibodies (B).

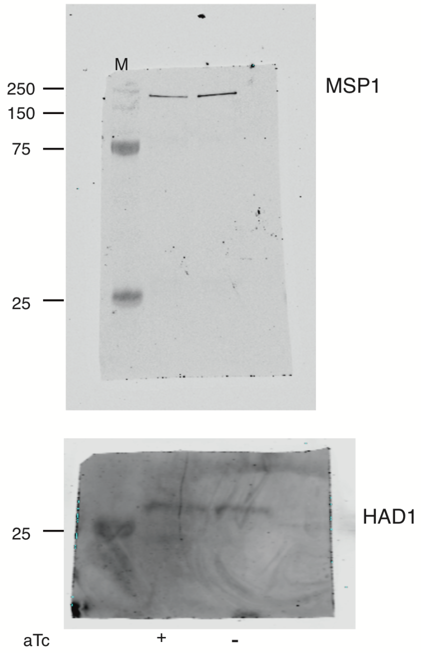

Figure S6. PMX knockdown results in accumulation of MSP1 precursor. Highly synchronous PMX^{apt} parasites were cultured to 44 hpi ±aTc and magnet-purified. Compound 1 was added at 1 uM for 4 hrs, washed out and parasites were left for 1 more hour. Extracts were prepared for western blot using whole schizont-infected erythrocytes. Immunoblot was performed with mAb anti-MSP1 12.4 and 9.8, which recognize full-length MSP1 at 193 kDa. MSP1 was quantified by densitometry, normalized to HAD1 loading control. MSP1 accumulation was 1.9±0.3 fold higher in the –aTc condition. Results are similar to those of Blackman and colleagues for inhibition of SUB1 (16).

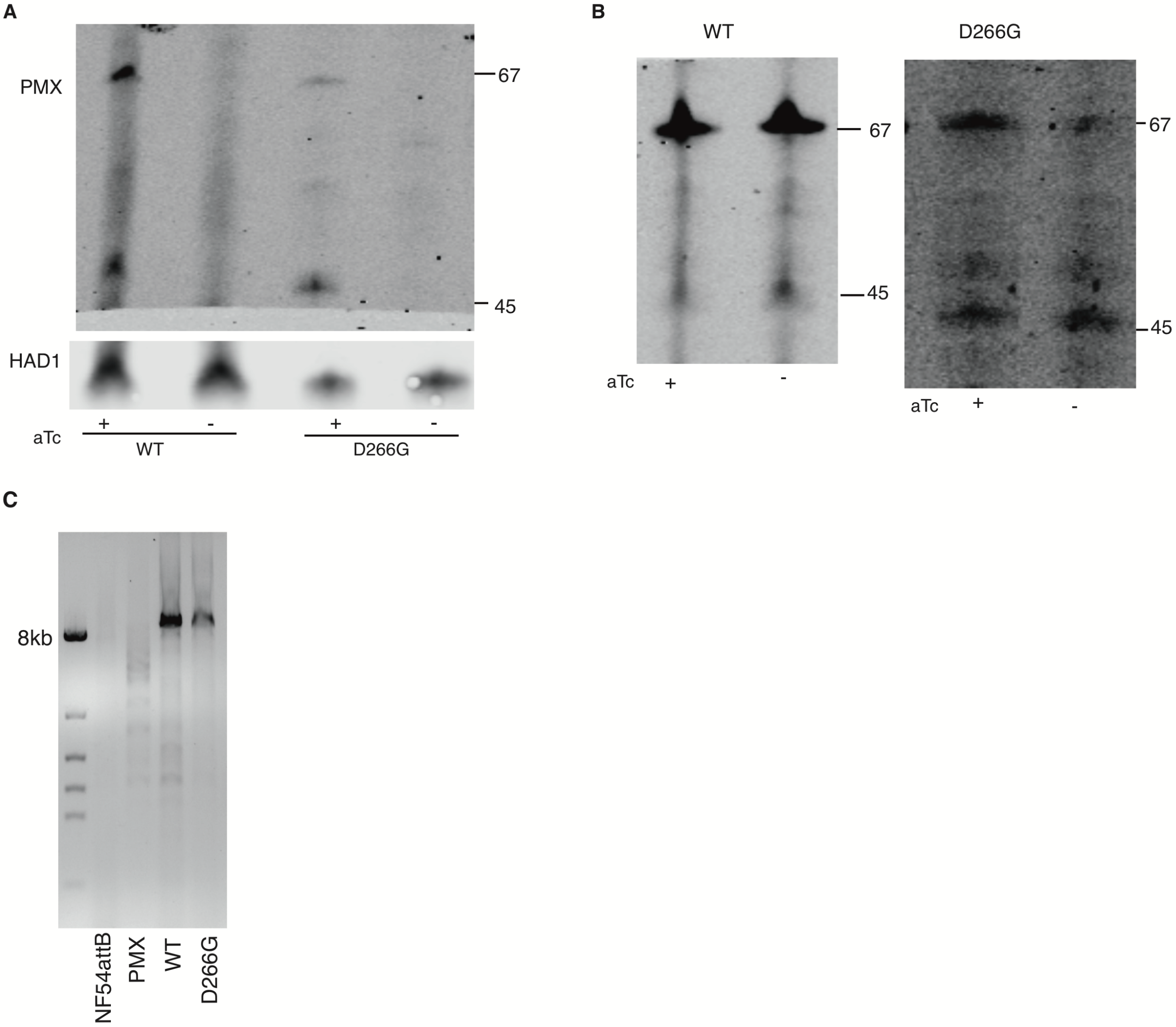

Fig. S7. Complementation of the PMX^{apt} line with WT and mutant second copies. (**A**) Anti-flag immunoblot showing KD of PMX expression upon aTc withdrawal from WT and D266G complementation strains. Ring-stage parasites were cultured +/- aTc and mature schizonts were prepared for blotting using anti-Flag antibodies (**B**) Expression of second copy WT and D266G proteins. Lysate from (A) was probed with anti-Myc antibodies. Pro and mature forms are marked on right. (**C**) PCR confirming integration of plasmid constructs encoding WT and D266G complementation copies of PMX. PCR was done using one primer on the chromosome near the Cg6 locus and a primer on our integrated plasmid. Expected PCR product size = ~10kb

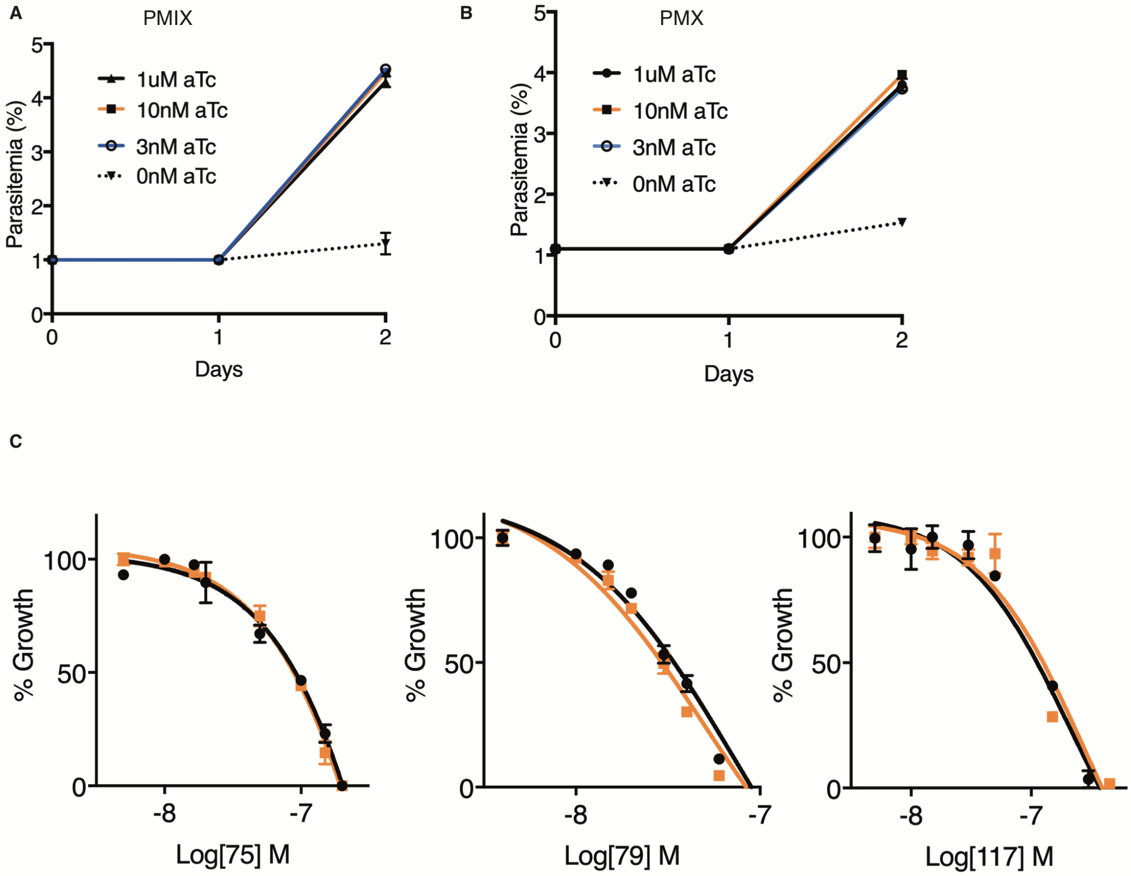

Fig. S8. aTc titration growth curves for PMIX^{apt} and PMX^{apt}. Representative growth curves for PMIX^{apt} (**A**) and PMX^{apt} (**B**) parasites over one cycle used for drug assays. Synchronous ring stage parasites were grown at the indicated aTc concentrations and the parasitemia was determined by flow cytometry. Western blots showing KD of protein expression are shown in Fig. 1B. (**C**) There is no shift in EC_{50} between 1μM (black) and 10nM aTc (orange) on the PMX^{apt} line. (In A; compared to 1uM aTc, t-test, $p<0.0001$ for 0nM aTc. In B; compared to 1uM aTc, t-test, $p<0.0001$ for 0nM aTc)

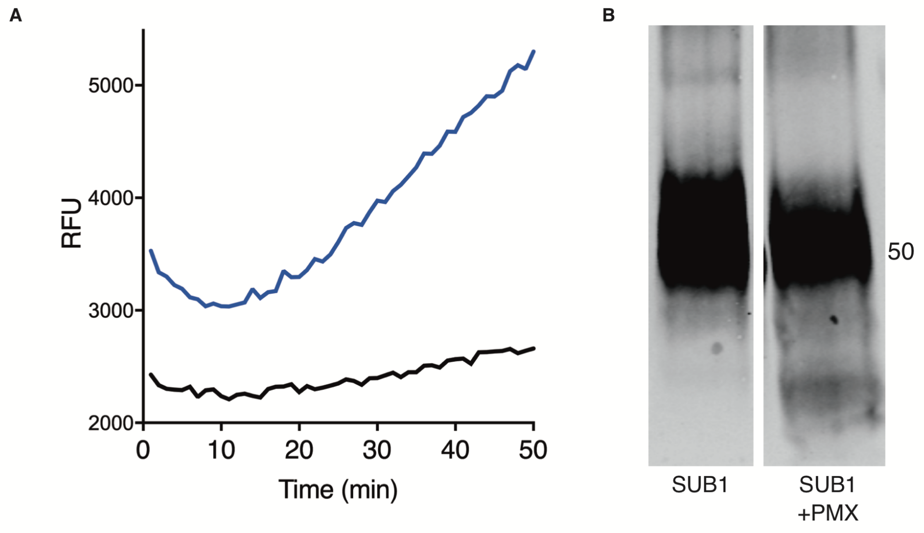

Fig. S9. PMX in vitro assays. (**A**) Progress curves for reaction of PMX with fluorogenic substrate (Blue curve is PMX, black curve is no PMX media control). (**B**) Cleavage of SUB1 by PMX *in vitro*. Full-length recombinant SUB1 was expressed and was isolated as the p54 form that is generated by autoprocessing during biosynthesis (18). SUB1 was incubated with recombinant PMX at pH 5.0 and cleavage was assessed by western blot. Control incubations with CWHM-117 (10 uM) gave no cleavage (not shown).

References and Notes

1. M. J. Meyers, D. E. Goldberg, Recent advances in plasmepsin medicinal chemistry and implications for future antimalarial drug discovery efforts. *Curr. Top. Med. Chem.* 12, 445–455 (2012). doi:10.2174/156802612799362959 Medline

2. C. Boss, S. Richard-Bildstein, T. Weller, W. Fischli, S. Meyer, C. Binkert, Inhibitors of the *Plasmodium falciparum* parasite aspartic protease plasmepsin II as potential antimalarial agents. *Curr. Med. Chem.* **10**, 883–907 (2003). doi:10.2174/0929867033457674 Medline

3. D. Rasina, M. Otikovs, J. Leitans, R. Recacha, O. V. Borysov, I. Kanepe-Lapsa, I. Domraceva, T. Pantelejevs, K. Tars, M. J. Blackman, K. Jaudzems, A. Jirgensons, Fragment-based discovery of 2-aminoquinazolin-4(3H)-ones as novel class nonpeptidomimetic inhibitors of the plasmepsins I, II, and IV. *J. Med. Chem.* **59**, 374–387 (2016). doi:10.1021/acs.jmedchem.5b01558 Medline

4. P. Bhaumik, A. Gustchina, A. Wlodawer, Structural studies of vacuolar plasmepsins. *Biochim. Biophys. Acta* **1824**, 207–223 (2012). doi:10.1016/j.bbapap.2011.04.008 Medline

5. J. A. Bonilla, T. D. Bonilla, C. A. Yowell, H. Fujioka, J. B. Dame, Critical roles for the digestive vacuole plasmepsins of *Plasmodium falciparum* in vacuolar function. *Mol. Microbiol.* **65**, 64–75 (2007). doi:10.1111/j.1365-2958.2007.05768.x Medline

6. R. Banerjee, J. Liu, W. Beatty, L. Pelosof, M. Klemba, D. E. Goldberg, Four plasmepsins are active in the *Plasmodium falciparum* food vacuole, including a protease with an active-site histidine. *Proc. Natl. Acad. Sci. U.S.A.* **99**, 990–995 (2002). doi:10.1073/pnas.022630099 Medline

7. I. Russo, S. Babbitt, V. Muralidharan, T. Butler, A. Oksman, D. E. Goldberg, Plasmepsin V licenses *Plasmodium* proteins for export into the host erythrocyte. *Nature* **463**, 632–636 (2010). doi:10.1038/nature08726 Medline

8. J. A. Boddey, A. N. Hodder, S. Günther, P. R. Gilson, H. Patsiouras, E. A. Kapp, J. A. Pearce, T. F. de Koning-Ward, R. J. Simpson, B. S. Crabb, A. F. Cowman, An aspartyl protease directs malaria effector proteins to the host cell. *Nature* **463**, 627–631 (2010). doi:10.1038/nature08728 Medline

9. A. N. Hodder, B. E. Sleebs, P. E. Czabotar, M. Gazdik, Y. Xu, M. T. O'Neill, S. Lopaticki, T. Nebl, T. Triglia, B. J. Smith, K. Lowes, J. A. Boddey, A. F. Cowman, Structural basis for plasmepsin V inhibition that blocks export of malaria proteins to human erythrocytes. *Nat. Struct. Mol. Biol.* **22**, 590–596 (2015). doi:10.1038/nsmb.3061 Medline

10. G. H. Coombs, D. E. Goldberg, M. Klemba, C. Berry, J. Kay, J. C. Mottram, Aspartic proteases of *Plasmodium falciparum* and other parasitic protozoa as drug targets. *Trends Parasitol.* **17**, 532–537 (2001). doi:10.1016/S1471-4922(01)02037-2 Medline

11. M. J. Meyers, M. D. Tortorella, J. Xu, L. Qin, Z. He, X. Lang, W. Zeng, W. Xu, L. Qin, M. J. Prinsen, F. M. Sverdrup, C. S. Eickhoff, D. W. Griggs, J. Oliva, P. G. Ruminski, E. J. Jacobsen, M. A. Campbell, D. C. Wood, D. E. Goldberg, X. Liu, Y. Lu, X. Lu, Z. Tu,

X. Lu, K. Ding, X. Chen, Evaluation of aminohydantoins as a novel class of antimalarial agents. *ACS Med. Chem. Lett.* **5**, 89–93 (2013). doi:10.1021/ml400412x Medline

12. S. M. Ganesan, A. Falla, S. J. Goldfless, A. S. Nasamu, J. C. Niles, Synthetic RNA-protein modules integrated with native translation mechanisms to control gene expression in malaria parasites. *Nat. Commun.* **7**, 10727 (2016). doi:10.1038/ncomms10727 Medline
13. S. Glushakova, D. Yin, N. Gartner, J. Zimmerberg, Quantification of malaria parasite release from infected erythrocytes: Inhibition by protein-free media. *Malar. J.* **6**, 61 (2007). doi:10.1186/1475-2875-6-61 Medline
14. P. Sharma, C. E. Chitnis, Key molecular events during host cell invasion by Apicomplexan pathogens. *Curr. Opin. Microbiol.* **16**, 432–437 (2013). doi:10.1016/j.mib.2013.07.004 Medline
15. S. Yeoh, R. A. O'Donnell, K. Koussis, A. R. Dluzewski, K. H. Ansell, S. A. Osborne, F. Hackett, C. Withers-Martinez, G. H. Mitchell, L. H. Bannister, J. S. Bryans, C. A. Kettleborough, M. J. Blackman, Subcellular discharge of a serine protease mediates release of invasive malaria parasites from host erythrocytes. *Cell* **131**, 1072–1083 (2007). doi:10.1016/j.cell.2007.10.049 Medline
16. K. Koussis, C. Withers-Martinez, S. Yeoh, M. Child, F. Hackett, E. Knuepfer, L. Juliano, U. Woehlbier, H. Bujard, M. J. Blackman, A multifunctional serine protease primes the malaria parasite for red blood cell invasion. *EMBO J.* **28**, 725–735 (2009). doi:10.1038/emboj.2009.22 Medline
17. D. Giganti, A. Bouillon, L. Tawk, F. Robert, M. Martinez, E. Crublet, P. Weber, C. Girard-Blanc, S. Petres, A. Haouz, J. F. Hernandez, O. Mercereau-Puijalon, P. M. Alzari, J. C. Barale, A novel *Plasmodium*-specific prodomain fold regulates the malaria drug target SUB1 subtilase. *Nat. Commun.* **5**, 4833 (2014). doi:10.1038/ncomms5833 Medline
18. M. Sajid, C. Withers-Martinez, M. J. Blackman, Maturation and specificity of *Plasmodium falciparum* subtilisin-like protease-1, a malaria merozoite subtilisin-like serine protease. *J. Biol. Chem.* **275**, 631–641 (2000). doi:10.1074/jbc.275.1.631 Medline
19. C. Withers-Martinez, J. W. Saldanha, B. Ely, F. Hackett, T. O'Connor, M. J. Blackman, Expression of recombinant *Plasmodium falciparum* subtilisin-like protease-1 in insect cells. Characterization, comparison with the parasite protease, and homology modeling. *J. Biol. Chem.* **277**, 29698–29709 (2002). doi:10.1074/jbc.M203088200 Medline
20. S. Arastu-Kapur, E. L. Ponder, U. P. Fonović, S. Yeoh, F. Yuan, M. Fonović, M. Grainger, C. I. Phillips, J. C. Powers, M. Bogyo, Identification of proteases that regulate erythrocyte rupture by the malaria parasite *Plasmodium falciparum*. *Nat. Chem. Biol.* **4**, 203–213 (2008). doi:10.1038/nchembio.70 Medline
21. R. Stallmach, M. Kavishwar, C. Withers-Martinez, F. Hackett, C. R. Collins, S. A. Howell, S. Yeoh, E. Knuepfer, A. J. Atid, A. A. Holder, M. J. Blackman, *Plasmodium falciparum* SERA5 plays a non-enzymatic role in the malarial asexual blood-stage lifecycle. *Mol. Microbiol.* **96**, 368–387 (2015). doi:10.1111/mmi.12941 Medline
22. F. J. Gamo, L. M. Sanz, J. Vidal, C. de Cozar, E. Alvarez, J. L. Lavandera, D. E. Vanderwall, D. V. Green, V. Kumar, S. Hasan, J. R. Brown, C. E. Peishoff, L. R. Cardon,

J. F. Garcia-Bustos, Thousands of chemical starting points for antimalarial lead identification. *Nature* **465**, 305–310 (2010). doi:10.1038/nature09107 Medline

23. V. Muralidharan, A. Oksman, P. Pal, S. Lindquist, D. E. Goldberg, *Plasmodium falciparum* heat shock protein 110 stabilizes the asparagine repeat-rich parasite proteome during malarial fevers. *Nat. Commun.* **3**, 1310 (2012). doi:10.1038/ncomms2306 Medline

24. M. Klemba, W. Beatty, I. Gluzman, D. E. Goldberg, Trafficking of plasmepsin II to the food vacuole of the malaria parasite *Plasmodium falciparum*. *J. Cell Biol.* **164**, 47–56 (2004). doi:10.1083/jcb200307147 Medline

25. N. J. Spillman, J. R. Beck, S. M. Ganesan, J. C. Niles, D. E. Goldberg, The chaperonin TRiC forms an oligomeric complex in the malaria parasite cytosol. *Cell. Microbiol.* **19**, e12719 (2017). doi:10.1111/cmi.12719 Medline

26. E. S. Istvan, J. P. Mallari, V. C. Corey, N. V. Dharia, G. R. Marshall, E. A. Winzeler, D. E. Goldberg, Esterase mutation is a mechanism of resistance to antimalarial compounds. *Nat. Commun.* **8**, 14240 (2017). doi:10.1038/ncomms14240 Medline

27. L. J. Nkrumah, R. A. Muhle, P. A. Moura, P. Ghosh, G. F. Hatfull, W. R. Jacobs Jr., D. A. Fidock, Efficient site-specific integration in *Plasmodium falciparum* chromosomes mediated by mycobacteriophage Bxb1 integrase. *Nat. Methods* **3**, 615–621 (2006). doi:10.1038/nmeth904 Medline

28. S. H. Adjalley, G. L. Johnston, T. Li, R. T. Eastman, E. H. Ekland, A. G. Eappen, A. Richman, B. K. Sim, M. C. Lee, S. L. Hoffman, D. A. Fidock, Quantitative assessment of *Plasmodium falciparum* sexual development reveals potent transmission-blocking activity by methylene blue. *Proc. Natl. Acad. Sci. U.S.A.* **108**, E1214–E1223 (2011). doi:10.1073/pnas.1112037108 Medline

29. H. M. Taylor, L. McRobert, M. Grainger, A. Sicard, A. R. Dluzewski, C. S. Hopp, A. A. Holder, D. A. Baker, The malaria parasite cyclic GMP-dependent protein kinase plays a central role in blood-stage schizogony. *Eukaryot. Cell* **9**, 37–45 (2010). doi:10.1128/EC.00186-09 Medline
